# The BSCM score: a guideline for surgical decision-making for brainstem cavernous malformations

**DOI:** 10.1007/s10143-021-01679-y

**Published:** 2021-10-29

**Authors:** Yang Yang, Julia Velz, Marian C. Neidert, Wei Lang, Luca Regli, Oliver Bozinov

**Affiliations:** 1 Department of Neurosurgery, Clinical Neuroscience Center, University Hospital of Zurich, University of Zurich, Ramistrasse 100, CH-8091 Zurich, Switzerland; 2 Department of Neurosurgery, Kantonsspital St. Gallen, Rorschacher Strasse 95, CH-9007 St. Gallen, Switzerland; 3 Department of Geriatric Medicine, University Hospital Zurich, City Hospital Waid Zurich, Tiechestrasse 99, CH-8037 Zurich, Switzerland

**Keywords:** Cavernous malformation, Brainstem, Resection, Grading system

## Abstract

Microsurgical resection of brainstem cavernous malformations (BSCMs) can be performed today with acceptable morbidity and mortality. However, in this highly eloquent location, the indication for surgery remains challenging. We aimed to elaborate a score system that may help clinicians with their choice of treatment in patients with BSCMs in this study. A single-center series of 88 consecutive BSCMs patients with 272 follow-up visits were included in this study. Univariable and multivariable generalized estimating equations (GEE) were constructed to identify the association of variables with treatment decisions. A score scale assigned points for variables that significantly contributed to surgical decision-making. Surgical treatment was recommended in 37 instances, while conservative treatment was proposed in 235 instances. The mean follow-up duration was 50.4 months, and the mean age at decision-making was 45.9 years. The mean BSCMs size was 14.3 ml. In the multivariable GEE model, patient age, lesion size, hemorrhagic event(s), mRS, and axial location were identified as significant factors for determining treatment options. With this proposed score scale (grades 0–XII), non-surgery was the first option at grades 0–III. The crossover point between surgery and non-surgery recommendations lay between grades V and VI while surgical treatment was found in favor at grades VII–X. In conclusion, the proposed BSCM operating score is a clinician-friendly tool, which may help neurosurgeons decide on the treatment for patients with BSCMs.

## Introduction

With the advances in microsurgical techniques and the theory of entry zones, surgical resection of brainstem cavernous malformations (BSCMs) can today be realized with acceptable morbidities and mortalities [1, 8, 9, 17, 21, 22, 25, 27]. However, surgical decision-making in patients with BSCMs remains a delicate balancing act. The surgical indication is often based on an individual surgeon’s judgment. The natural risk of (repetitive) hemorrhage with subsequent neurological decline needs to be weighed against the perioperative risks of microsurgical resection, given the complex anatomy and highly eloquent function of the brainstem.

Generally, conservative clinical management is recommended in patients who are asymptomatic with small lesions [2, 15]. Surgical resection is recommended for patients with symptomatic accessible lesions, repeated hemorrhages, and neurological decline [1, 17, 24]. However, for deep seated, especially ventrally located BSCMs, surgery is more controversial because of the relatively high morbidity and mortality rate [6, 25]. There is also no consensus on the timing of surgery in such patients. Existing publications suggested that patients can benefit from either immediate or subacute surgery after neurological deficits or hemorrhage events [4, 5, 8, 21]. Therefore, in this study, we intend to analyze the clinical decisions on BSCMs over the past decade in greater detail and devise a BSCM score scale. It is hypothesized that this score scale will help neurosurgeons decide on the best treatment time point in patients with BSCMs.

## Methods

### Study design and data collection

This study is a retrospective, single-center analysis of all patients with BSCMs who underwent conservative or surgical management between 2006 and 2018. The study was approved by the local Ethics Committee (KEK-ZH 2017–00,330). Patients with both a radiological or histological diagnosis of BSCMs and available follow-up data were included in this study. Patients with multiple intracranial CMs were also included when the BSCM was primarily responsible for the clinical symptoms and treatment considerations. The electronic patient chart was screened for patient baseline characteristics, lesion size and location, and neurological condition at each follow-up visit. Magnetic resonance imaging (MRI) was blind reviewed by two independent neurosurgeons.

### Study variables

Patient demographics included sex and age at the decision. The dates of admission, and of each follow-up, and the number of hemorrhagic events based on MRI were recorded. The lesion size was determined by the maximum diameter (in millimeters) on T2-weighted MRI or fluid-attenuated inversion recovery (FLAIR). The lesion location, as described in our previous study, was divided into three categories based on the position of the lesion core in the axial plane [28]: (i) median: the lesion is located on the axial midline; (ii) paramedian: the lesion is unilaterally located between the midline and lateral line; and (iii) lateral: the lesion core is located between the lateral line and extreme lateral line. Each category has a corresponding exophytic type. The classification applies regardless of whether the center of a lesion is in the midbrain, pons, or medulla [28]. A hemorrhagic event was defined as new intra-lesion or extra-lesion blood products on MRI, with or without a clear history of an acute neurological deficit [8]. Patients with a slowly progressive clinical course or only hemosiderin on MRI or both were not considered to have had a new hemorrhagic event [8]. As described in our previous study, we perform routine radiological and clinical follow-up of 3 months after initial diagnosis of BSCM and yearly in patients with BSCM managed conservatively [22, 23]. In cases of BSCM hemorrhages between follow-up intervals, patients were ultimately referred to our department (in patient clinic/emergency room). All follow-ups were done in person by a neurosurgeon [23]. The modified Rankin Scale (mRS) score was used to assess the degree of neurological impairment at each follow-up [20]. Treatment decision, either indication for conservative or surgical management, was reviewed for each patient at each follow-up based on the medical record. In cases where microsurgical resection of BSCMs was indicated, the surgery was conducted within 1 month.

Of note, we have recently shown based on our data that it appears reasonable to limit clinical management to patient education and symptom-driven follow-up strategy and thus to avoid unnecessary routine follow-up imaging [23].

### Statistical analysis

Descriptive statistics were expressed as mean ± standard error (SE) and percentage for continuous and categorical variables, respectively. Significant differences were analyzed by the *t-*test and chi-square test. Fisher’s exact test was used when appropriate.

A univariable generalized estimating equation (GEE) model tested the association between each variable and the surgical decision-making. A multivariable GEE was modeled for the association between combined variables and the treatment decision. Factors were weighted by β coefficient estimates in the final model. The threshold for the *p*-value is not strictly defined (*P* < 0.2) for including predictors in the multivariable GEE model, because of the inaccurate control of potential confounders by using bivariable selection [19]. Significant variables were assigned points to create a treatment grading system. Based on this grading system, the total scores of each instance were then calculated to evaluate the distribution of treatment decisions. The data analysis, including the modeling of GEE, was performed by SAS software, version 9.4 (SAS Institute Inc., Cary, NC, USA) [12].

## Results

### Demographics and lesion characteristics

A total of 118 patients were retrieved from the database, and 88 patients with 272 follow-up visits were included in this study. Surgical resection was recommended in 37 instances, while non-surgical management was proposed in 235 instances. The mean ages at decision-making in the surgical and non-surgical decision-making groups were 40.4 and 46.8 years, respectively (Table 1).

**Table 1:** Demographics, lesion characteristics by treatment decisions

Variable	Total/average	Surgery	Non-surgery	P Value
Overall	272	37	235	-
Follow-up duration (months)	50.4 ± 4.1	31.9 ± 10.6	53.3 ± 4.5	0.08
Age at decision-making (years)	45.9 ± 1.0	40.4 ± 2.4	46.8 ± 1.1	0.03
Sex				0.07
Female (%)	47 (53.4)	25 (67.6)	34 (49.3)	
Male (%)	41 (46.6)	12 (32.4)	35 (50.7)	
Lesion size (mm)	14.3 ± 0.4	19.6 ± 1.4	13.5 ± 0.4	< 0.001
Axial location				0.81
Median (%)	41 (15.1)	6 (13.5)	36 (15.3)	
Paramedian (%)	171 (62.9)	25 (67.6)	146 (62.1)	
Lateral (%)	60 (22.0)	7 (18.9)	53 (22.6)	
Exophytic (%)				0.70^*^
Yes	14 (5.1)	1 (2.7)	13 (5.5)	
No	258 (94.9)	36 (97.3)	222 (94.5)	
Hemorrhagic event				< 0.001^*^
0	53 (19.5)	1 (2.7)	52 (22.1)	
1	168 (61.8)	16 (43.2)	152 (64.7)	
2	38 (14.0)	16 (43.2)	22 (9.4)	
3	11 (4.0)	4 (10.8)	7 (3.0)	
4	2 (0.7)	0 (0.0)	2 (0.8)	
mRS at decision				< 0.001^*^
0	72 (26.5)	1 (2.7)	71 (30.2)	
1	103 (37.9)	13 (35.1)	90 (38.3)	
2	68 (25.0)	15 (40.5)	53 (22.6)	
3	21 (7.4)	5 (13.5)	16 (6.8)	
4	6 (2.6)	1 (2.7)	5 (2.1)	
5	2 (0.7)	2 (5.4)	0 (0.0)	

^*^Fisher’s exact test

The mean lesion size in the surgical decision-making group was significantly larger than that in the non-surgical group (19.6 versus 13.5 mm, *P* < 0.001). In 204 (86.8%) instances, conservative treatment was proposed in patients with one or no hemorrhagic event. In 161 (68.5%) instances, conservative management was proposed in patients with mRS 0–1. In 20 (54.0%) instances, microsurgical resection was recommended in patients with two or more hemorrhagic events. In 23 (62.1%) instances, microsurgical resection was recommended in patients with mRS of 2–5 (Table 1).

### Generalized estimating equation (GEE) analysis

Univariable GEE analysis identified lesion size (*P* ≤ 0.07), mRS (*P* ≤ 0.01), and hemorrhagic event (*P* ≤ 0.09) as factors likely to increase the odds ratio of surgical decision-making, while being over 60 years old at decision-making (*P* ≤ 0.17) was associated with a decrease of the odds ratio. There was no association observed between lesion location nor respectively exophytic type and treatment recommendation (Table 2). In the multivariable GEE model, being over 60 years old, a lesion size of over 10 mm, hemorrhagic event(s), mRS, and no midline crossing were identified to be significant factors in determining treatment option (Table 3).

**Table 2:** Univariable generalized estimating equation (GEE) analysis

Variable	OR	95% CI	*P* value
^*^Age at decision-making (years)			
> 20, ≤ 30	− 0.14	− 1.64–1.35	0.85
> 30, ≤ 40	− 0.29	− 1.67–1.10	0.68
> 40, ≤ 50	− 0.39	− 1.80–1.02	0.59
> 50, ≤ 60	− 0.77	− 2.19–0.65	0.29
> 60, ≤ 70	− 1.11	− 2.68–0.46	0.17
> 70	− 1.87	− 4.02–0.28	0.09
^†^Size (mm)			
> 10, ≤ 20	0.99	− 0.08–2.05	0.07
> 20, ≤ 30	2.15	0.97–3.33	< 0.001
> 30	3.31	1.01–5.61	0.005
Axial location	0.30	− 0.6–1.07	0.44
Exophytic	− 0.46	− 2.63–1.71	0.68
^#^mRS			
mRS = 1	2.20	0.44–3.99	0.01
mRS = 2	2.66	0.85–4.48	0.004
mRS = 3	2.65	0.74–4.55	0.01
mRS = 4, 5	3.65	0.78–6.51	0.01
^§^Hemorrhagic event (HE)			
HE = 1	1.49	− 0.25–3.23	0.09
HE = 2	3.31	1.46–5.17	< 0.001
HE ≥ 3	3.25	1.34–5.17	< 0.001

^*^The baseline reference is age ≤ 20 years old. ^†^The baseline reference is size ≤ 10 mm. ^#^The baseline reference is mRS = 0. ^§^The baseline reference is HE = 0

**Table 3:** Multivariable GEE model

Variable	OR	95% CI	*P* value
Intercept	− 7.70	− 10.54 to − 4.85	< 0.001
^*^Size (mm)			
> 10, ≤ 20	1.18	0.03–2.34	0.04
> 20, ≤ 30	1.99	0.77–3.21	0.001
> 30	4.89	2.68–7.11	< 0.001
^†^Age at decision-making (years)			
> 60, ≤ 70	-1.59	− 3.82–0.63	0.16
> 70	-3.25	− 5.32 to − 1.18	0.002
^#^mRS			
mRS = 1	2.53	0.14–4.91	0.04
mRS = 2	2.44	0.12–4.76	0.04
mRS = 3	2.58	0.06–5.11	0.05
mRS ≥ 4	2.88	− 0.53–6.28	0.10
^§^Hemorrhagic event (HE)			
HE = 1	2.20	− 0.03–4.42	0.06
HE = 2	4.09	1.94–6.23	< 0.001
HE ≥ 3	3.01	0.75–5.27	0.009
Crossing midline			
No	0.93	− 0.22–2.07	0.11

^*^The baseline reference is size ≤ 10 mm

^†^The baseline reference is age ≤ 20 years old

^#^The baseline reference is mRS = 0

^§^The baseline reference is HE = 0

### Proposed score system

The grading system for the BSCM treatment decision was constructed using the decision-related factors in the multivariable GEE model. Scores were assigned for each of the factors as shown in Table 4. A total of 13 grades (0–XII) were defined. Grade (0–XII) = maximum size (0–3) + mRS (0–4) + hemorrhagic events (0–3) + age at decision-making (0–1) + crossing midline or not (0–1).

**Table 4:** Points assigned to each factor of the score scale

Size (mm)	Points	^#^HE (time)	Points
≤ 10	0	0	0
> 10, ≤ 20	1	1	1
> 20, ≤ 30	2	2	2
> 30	3	≥ 3	3
mRS (0–5)	Points	Age (years)	Points
0	0	> 60	0
1	1	≤ 60	1
2	2	Crossing midline	points
3	3	Yes	0
4–5	4	No	1

^#^*HE*, hemorrhagic event

**Fig. 1:**
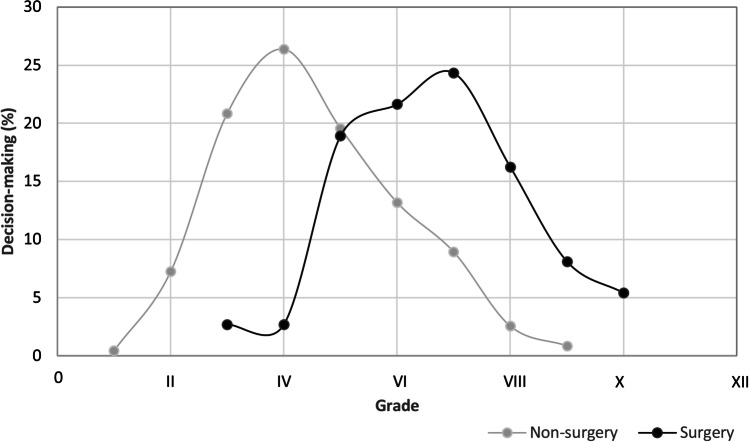
Treatment options using the score scale. The percentage of non-surgical decision-making begins at grade I and reaches a peak at grade IV. It then decreases and ends at grade IX. The percentage of surgical decision-making begins at grade III, sharply increases after grade IV, and peaks at grade VII before decreasing and ending at grade X. The crossover point between surgical and conservative management decision-making percentage of each group lies between grades V and VI

Table 5 and Fig. 1 show the distributions of treatment decisions with the proposed BSCM grading system. The percentage of non-surgical decision-making began at grade I, reached its peak at grade IV then decreased before ending at grade IX. The percentage of surgical decision-making began at grade III, sharply increased after grade IV, peaked at grade VII, before decreasing and ending at grade X (Fig. 1). The crossover point between surgical and conservative management decision-making was found between grades V and VI.

**Table 5:** Treatment option according to the score scale

Grade	Surgical decision-making		Non-surgical decision-making	
	*N*	Percentage	*N*	Percentage
0	0	0	0	0
I	0	0	1	0.4
II	0	0	17	7.2
III	1	2.7	49	20.8
IV	1	2.7	62	26.4
V	7	18.9	46	19.6
VI	8	21.6	31	13.2
VII	9	24.3	21	9.0
VIII	6	16.2	6	2.6
IX	3	8.1	2	0.8
X	2	5.4	0	0
XI	0	0	0	0
XII	0	0	0	0
Total	37	100	235	100

## Discussion

Surgical decision-making for patients with BSCMs, including surgical indications and timing, is often discussed without consensus. There currently exists only one grading scale to predict the postoperatively neurological outcome of BSCMs [8]. While that grading system is easy to apply in practice and helps to predict surgical outcomes in BSCM patients, it may have certain limitations for surgical decision-making. In that grading system, the score of lesion size is assigned to 0 or 1 based on a cutoff point of 20 mm in the axial plane. However, some giant BSCMs may merit a higher priority for surgical resection. In the present study, we summarized the patients’ baseline and lesion characteristics with regard to treatment recommendations. Results of the multivariable GEE model reveal that lesion size, neurological status, hemorrhagic events, age at the decision, and crossing axial midline are all associated with the treatment recommendations. Lesion size over 30 mm is the strongest indication for surgical decision-making. Based on the GEE analysis, we developed a score system for the surgical decision-making of BSCM.

### Lesion size

Size is a vital element for considering surgery of intracranial lesions. In the Spetzler-Martin grading system for surgical decision-making in patients with brain AVM, the lesion size is divided into three subgroups: small (< 3 cm), medium (3–6 cm), and large (> 6 cm) [18]. In BSCM patients, Garcia et al. [8] propose the grading system to predict the surgical outcome, in which size is dichotomized by 20 mm. In the present study, the maximal diameter is also shown to be an important factor in the treatment recommendation.

It is still unclear whether lesion size increases the risk of cavernoma hemorrhage. Some studies suggest that larger lesions are associated with higher primary and recurrent hemorrhage rates [3, 13, 15]. On the other hand, some researchers report that lesion size does not significantly affect hemorrhage risk [10, 14]. In the final GEE model, covariation of these two factors is not significant in our analysis. It should be considered, however, that despite the unresolved question of the influence of size on the bleeding risk, there is likely to be a natural tendency for a surgeon to decide on surgery in larger lesions. This may directly influence our data (larger lesions in the surgical group).

### Neurological status and hemorrhagic event

In the present study, the patient’s neurological status (evaluated by mRS) and any hemorrhagic events are included in the grading system. This is in line with the literature, in which neurological decline and bleeding event(s) have been identified as major factors in the consideration of microsurgical resection of BSCMs [1, 9, 11, 17, 26, 29]. Recently, Xie et al. [26] have recommended that microsurgical resection of BSCMs should be performed in patients with severe neurological deficits (mRS 4 or 5), even though approximately 74% of these patients have no signs of gradual recovery. In our study, we have a small sample size of patients with severe neurological deficits (mRS = 4, 5), which cannot be analyzed separately. Therefore, patients with mRS of 4 and 5 are allocated into one subgroup for analysis and assigned the same score point in the final grading scale.

It is reported that two-thirds of BSCMs hemorrhages can cause clinical symptoms [11]. A previous hemorrhagic event can be associated with as much as a sevenfold increase in the risk of rebleeding [1]. Hauck et al. [11] recommend early resection of the lesion after the first hemorrhagic event, considering the high risk of recurrent events and the patient’s preoperative condition as strong predictors of the overall outcome. Tsuji et al. [21] also suggest early surgery considering the remarkable decrease in postoperative rebleeding rate. Our analysis reveals that surgery is proposed in 33 instances (89.1%) with ≤ 2 hemorrhagic events. The second hemorrhagic event is the strongest indication for surgical decision-making. In the case of severe neurological deficits, which has a high morbidity rate in all studies, our proposed guideline should help justify such an intervention. Regarding those patients without severe neurological deficits after the first hemorrhage, conservative treatment and follow-up visits are suggested, which is also reflected in the treatment recommendation in grade III or IV (Fig. 1).

### Age at a decision and axial location

Age is a prognostic factor in the grading system for predicting the postoperative neurological outcome of BSCMs [8]. In the present study, significance is found for patients over 60 years in the regression model, after stratifying by 10 years. The result is in line with clinical practice, where the age of an adult is usually less likely to be a factor in surgical decision-making, except when the patient is over the age of 60.

Based on our data, surgical resection is seldom recommended for BSCMs crossing the axial midline, which is supported by the emerging evidence that midline crossing in the axial plane is a significant predictor for a worse outcome [8]. However, it should be noted that lesion location, in some other studies, is neither associated with a higher risk of a hemorrhagic event in BSCMs [7, 16] nor with a surgical outcome in midbrain CMs [21]. The axial location is significant in the multivariable GEE model, although not in univariable GEE analysis, which might be interpreted as the small sample size of the surgical decision-making or potential bias in the retrospective analysis.

### Treatment options under this grading scale

**Fig. 2:**
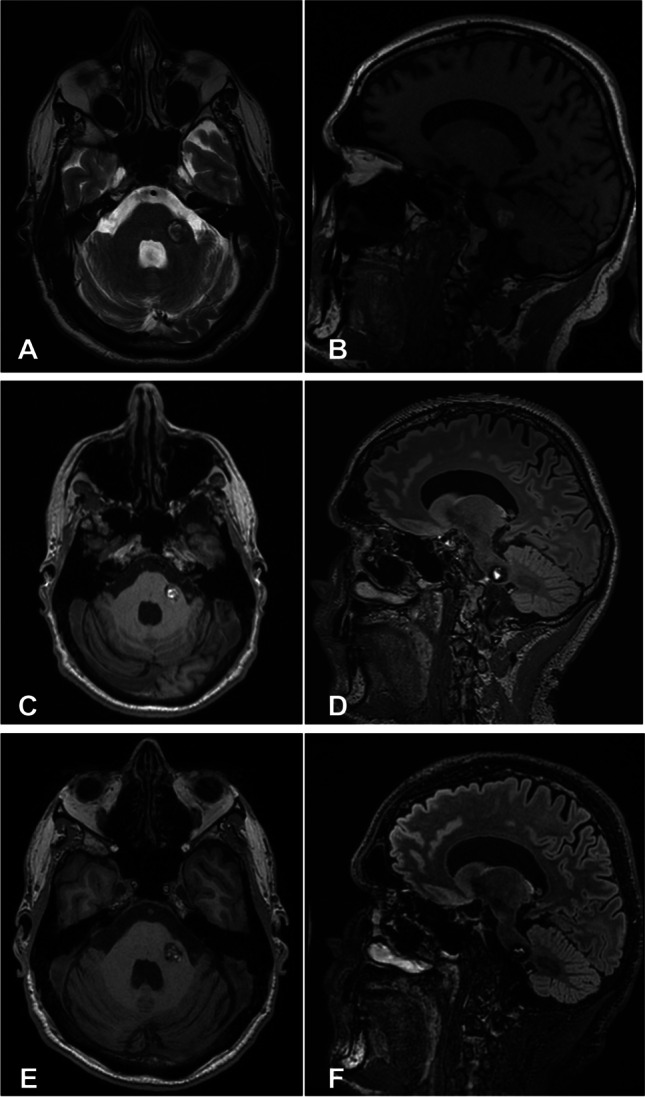
A 41-year-old man presented with repetitive hypesthesia in the anterior left tongue. MRI scan confirmed a 16 × 17 × 13 mm BSCM in the pontocerebellar (**A** and **B**). The BSCM score was grade V (size = 1, mRS = 1, hemorrhage event = 1, age = 1, and no midline crossing = 1), and conservative management was recommended. After 2 months, a follow-up MRI scan showed no further progression or fresh bleeding (**C** and **D**). The operation score was unchanged, and we recommended the patient for annual follow-up. The last follow-up was 4 years later, with no further deterioration of neurological status (**E** and **F**). The BSCM score remained at grade V

**Fig. 3:**
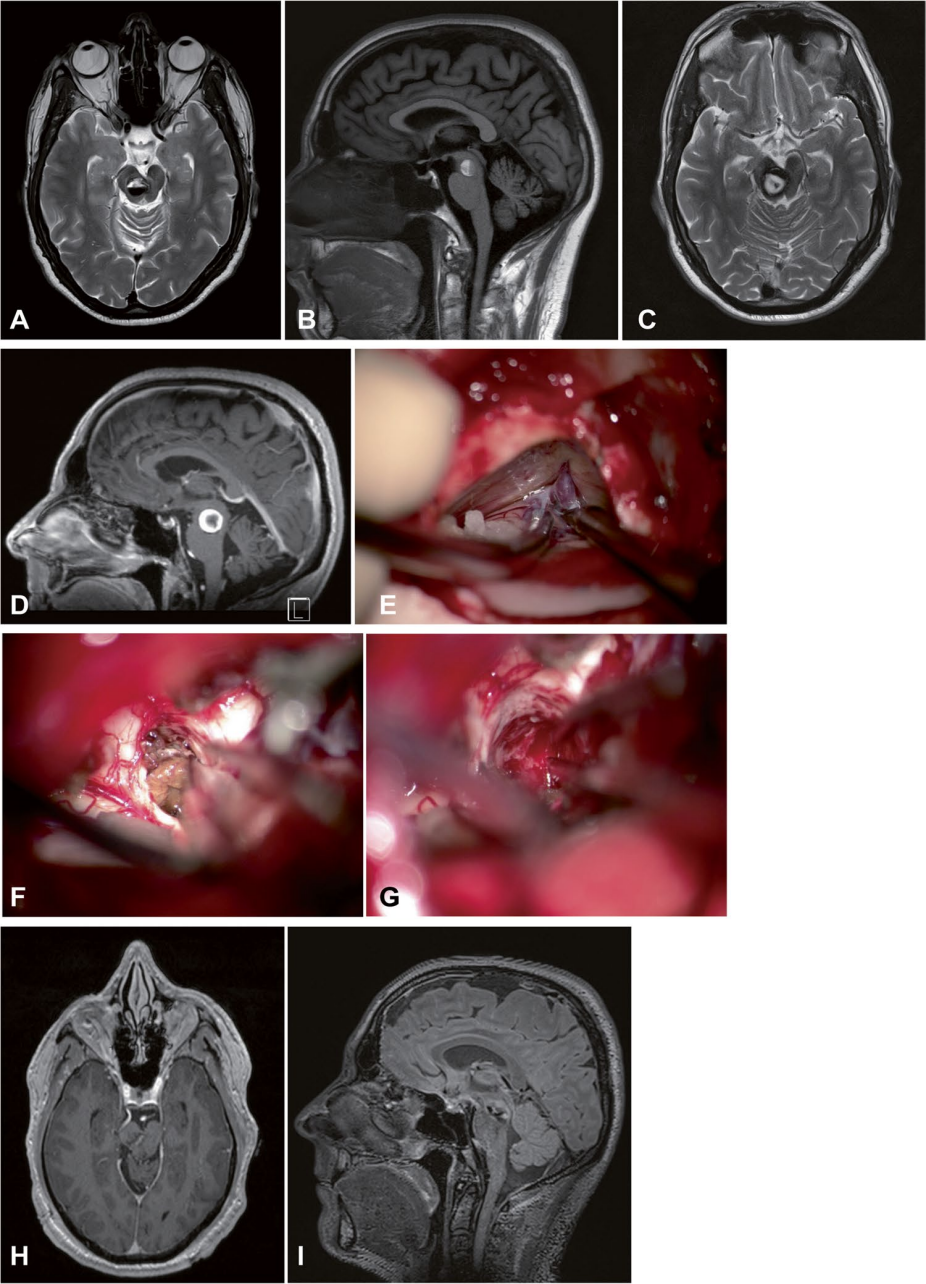
A 56-year-old man presented with diplopia, abduction weakness of the right eye, dysarthria, weakness in both hands. He was referred to our department after the first diagnosis of BSCM bleeding in another hospital. The MRI scan revealed a 19 × 18 × 13 mm BSCM in the ponto-mesencephalon (**A** and **B**). The BSCM score was grade VI (size = 1, mRS = 2, hemorrhage event = 1, age = 1, and no midline crossing = 1), and conservative treatment was suggested. Two weeks later, the patient had another BSCM bleeding without an increase in lesion size or deterioration of neurological deficits (**C** and **D**). The BSCM score was grade VII. Surgical resection of BSCM was performed with a retrosigmoid approach (**E**, **F**, and **G**). Postoperative MRI confirmed the total resection (**H** and **I**). The patient was neurologically intact at the last follow-up one and a half years later

**Fig. 4:**
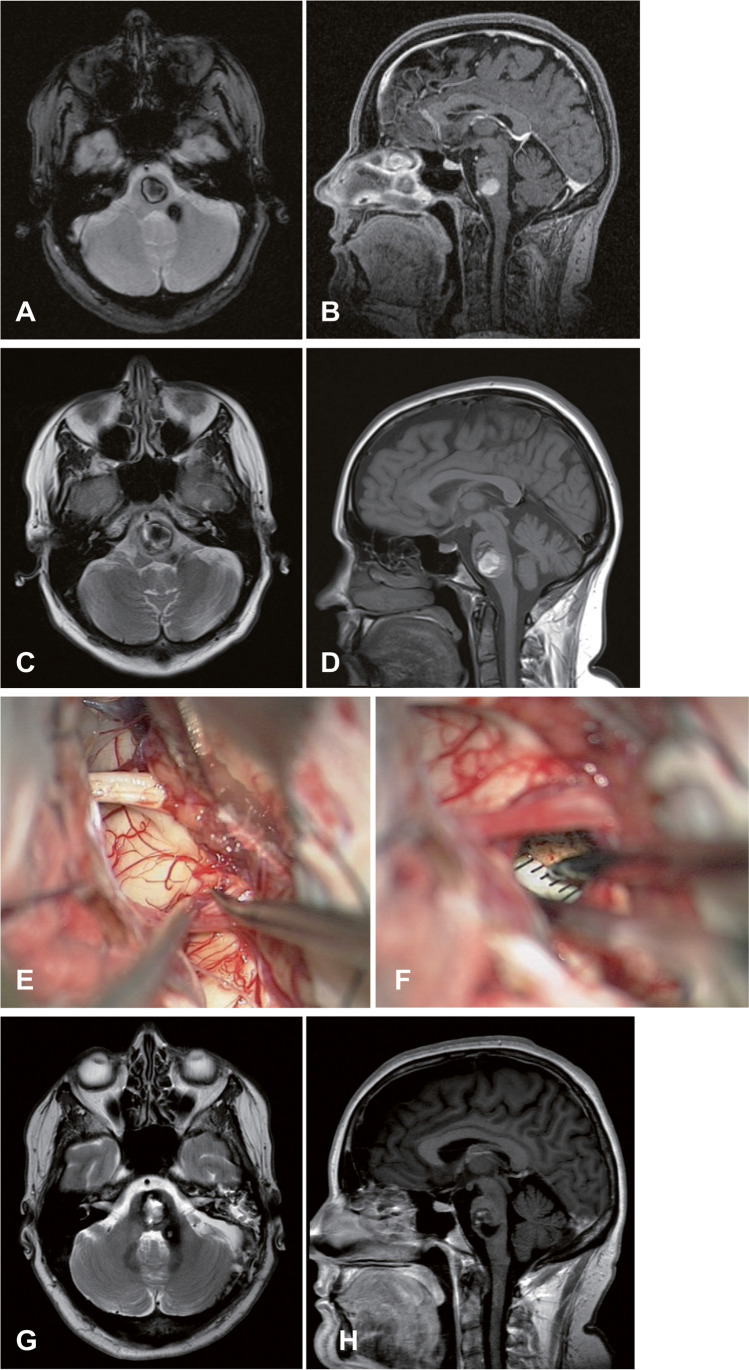
A 44-year-old woman, diagnosed with pontine CM in another hospital due to a hemorrhage event 8 years previously, was referred to our department with dysesthesia of the tongue and fingertips. The MRI scan confirmed multiple intracranial CMs with one large (15 × 21 × 22 mm) in the pons (**A** and **B**). The BSCM score was grade VI (size = 2, mRS = 1, hemorrhage event = 2, age = 1, and crossing midline = 0), and conservative treatment was recommended. Two months later, the patient had a recurrent hemorrhage in the pontine CM with right sensorimotor dysfunction, diplopia, and neuroimaging showed enlargement (**C** and **D**). The BSCM score was grade X (size = 2, mRS = 4, hemorrhage event = 3, age = 1, and crossing midline = 0). The suboccipital transcondylar approach was used for pontine CM resection (**E**, **F**). Postoperative MRI showed partial residual BSCM (**G** and **H**). The patient was neurologically intact at the last follow-up 2 years later

The grading scale is generalized from the multivariable GEE model. Under this scale, non-surgery is the first option at grades 0–II. Conservative treatment is still highly recommended at grade III, though the surgery was also already considered in one instance. There is a crossover point in the percentage of each group at grade VI (Table 5 and Fig. 1) and change of a total number in the decision for surgery at grades VIII to XI. The most appropriate recommendation at grades V and VI may become clearer when more clinical cases are enrolled for further validation. Surgical treatment should be the priority for patients in grades VII–X, although a downswing is observed in these grades because of fewer cases in general (Fig. 1). The relatively small case numbers in grades X–XII with unfavorable outcome suggest that these patients should be carefully selected for surgery. Figures 2, 3, and 4 are illustrative cases showing treatment options for BSCM patients in our clinical practice.

This study elaborates on the clinical decisions and the BSCM score scale based on the BSCM database over a 12-year period in one tertiary referral center. Despite the strong points, several factors need to be taken into account when evaluating the results of this study. The external validity of the proposed score scale demands further evaluation and validation given the retrospective nature of the study and the selection bias during the data collection. Moreover, every patient has a highly individual clinical course and condition. Personal needs should be fully considered as well together with the treatment option based on the score scale.

## Conclusion

The proposed BSCM grading scale is a clinician-friendly tool including all statistically relevant decision aspects, which may help neurosurgeons in BSCM management decide on the best treatment time point.

## Data Availability

The datasets generated for this study are available from the corresponding author on reasonable request.
